# Boosting Recovery During Sleep by Means of Auditory Stimulation

**DOI:** 10.3389/fnins.2022.755958

**Published:** 2022-02-02

**Authors:** Elena Krugliakova, Jelena Skorucak, Georgia Sousouri, Sven Leach, Sophia Snipes, Maria Laura Ferster, Giulia Da Poian, Walter Karlen, Reto Huber

**Affiliations:** ^1^Child Development Centre and Children’s Research Centre, University Children’s Hospital Zurich, University of Zurich, Zurich, Switzerland; ^2^Mobile Health Systems Lab, Department of Health Sciences and Technology, ETH Zürich, Zurich, Switzerland; ^3^Neural Control of Movement Lab, Department of Health Sciences and Technology, ETH Zürich, Zurich, Switzerland; ^4^Department of Child and Adolescent Psychiatry and Psychotherapy, Psychiatric Hospital, University of Zurich, Zurich, Switzerland

**Keywords:** auditory stimulation, non-rapid eye movement sleep (NREM), sleep homeostasis, recovery, slow waves

## Abstract

Sufficient recovery during sleep is the basis of physical and psychological well-being. Understanding the physiological mechanisms underlying this restorative function is essential for developing novel approaches to promote recovery during sleep. Phase-targeted auditory stimulation (PTAS) is an increasingly popular technique for boosting the key electrophysiological marker of recovery during sleep, slow-wave activity (SWA, 1–4 Hz EEG power). However, it is unknown whether PTAS induces physiological sleep. In this study, we demonstrate that, when applied during deep sleep, PTAS accelerates SWA decline across the night which is associated with an overnight improvement in attentional performance. Thus, we provide evidence that PTAS enhances physiological sleep and demonstrate under which conditions this occurs most efficiently. These findings will be important for future translation into clinical populations suffering from insufficient recovery during sleep.

## Introduction

The recovery function of sleep is essential. In case of insufficient recovery, we are tired and suffer from well-documented cognitive impairments, such as lapses in attention and reduced vigilance. Insufficient recovery during sleep can be caused by sleep disorders and other pathologies but can also be the result of curtailed sleep in healthy individuals (Van Dongen and Dinges, 2003).

According to the two-process model of sleep regulation, sleep is regulated by a circadian and a homeostatic process. While the circadian process depends on the time of the day and provides the ideal window for sleep, the homeostatic process depends on the duration of wake and sleep and reflects the build-up of sleep pressure (i.e., sleepiness) during the day and dissipation of sleep pressure (i.e., recovery) during sleep (Borbély et al., 1981; Dijk et al., 1990; Achermann and Borbély, 2003).

Slow-wave activity [SWA, power in delta frequency range (1–4 Hz) recorded by electroencephalography (EEG)] during non-rapid eye movement (NREM) sleep is a well-established electrophysiological correlate of the homeostatic regulation of sleep. As expected from such a correlate, (1) SWA is highest at the beginning of a night and this initial level is regulated in a dose-dependent way: The longer an individual is awake, the higher SWA is during sleep the following night and (2) SWA exponentially decreases throughout the night, reflecting the dissipation of sleep pressure, i.e., recovery during sleep. Consequently, (1) sleep restriction and sleep deprivation lead to increased levels of SWA associated with an initially faster dissipation of sleep pressure (Borbély, 1982) and (2) suppression of SWA during sleep by keeping participants in light sleep, slows down the dissipation of sleep pressure and thus hinders recovery during sleep with negative consequences on daytime sleepiness and cognitive processes (Gillberg and Åkerstedt, 1994; Dijk et al., 2006).

Accordingly, there is an increasing interest in sleep enhancement, i.e., the boosting of SWA. Pharmacological agents, as well as magnetic and electrical stimulation, have been investigated in terms of enhancing sleep by boosting SWA. Unfortunately, commonly used pharmacological sleep aids (benzodiazepines, and benzodiazepine analogs), though effective in initiating and consolidating sleep, have been shown to decrease SWA and, hence, are not considered to induce physiological sleep (Borbély et al., 1985). Gamma hydroxybutyrate (GHB) is a promising substance that induces SWA but can be problematic in terms of dependency and misuse (Dornbierer et al., 2019). Non-invasive brain stimulation, such as transcranial magnetic stimulation (TMS) and transcranial direct current stimulation (tDCS), has been shown to boost slow waves (Nitsche and Paulus, 2000; Marshall et al., 2004; Massimini et al., 2007), however, such technologies lack the outlook into a long-term application.

In recent years, phase-targeted auditory stimulation (PTAS) has become a popular tool to manipulate slow waves (Ngo et al., 2015). During PTAS brief (50 ms) pink noise stimuli (∼50 dB SPL) are presented in real-time locked to the phase of slow waves. This tool is suitable for long-term application because it is portable, non-invasive, and simple to apply (Ferster et al., 2019; Arnal et al., 2020; Grifantini, 2020). PTAS has already been recognized as a potential therapeutic intervention for several disorders in which sleep-dependent recovery is impaired such as attention-deficit hyperactivity disorder (Prehn-Kristensen et al., 2020) and mild cognitive impairment (Papalambros et al., 2019).

Although numerous studies have shown that slow waves can be promoted *via* PTAS, research has primarily focused on its effect on memory consolidation, and in rare cases on other health-related physiological parameters, like the immune-supportive hormonal milieu and autonomic function in sleep (Besedovsky et al., 2017; Grimaldi et al., 2019). To the best of our knowledge, the effect of PTAS on the recovery function of sleep is not yet examined. If indeed PTAS induces physiological sleep, we would expect a preservation of sleep homeostasis under stimulation. Specifically, according to the homeostatic regulation of sleep, boosting SWA in the first 2.5 h of sleep, thus promoting the dissipation of sleep pressure during the stimulation time, should lead to a negative rebound, i.e., lower SWA values, thereafter. Such a steeper decline of SWA across the night would indicate a faster dissipation of sleep pressure, i.e., a boost in sleep-dependent recovery.

A key limiting factor for developing tools promoting recovery during sleep is a knowledge gap between the regulation of SWA recorded from the scalp and the underlying neuronal activity during sleep and its functions. More specifically, we know that the thalamo-cortical system generates slow waves and EEG slow waves are reflected by large populations of cortical neurons synchronously oscillating between an active and a silent state (Steriade et al., 1993). Further, it is well established that slow waves travel across different brain regions (Massimini et al., 2004), and that they are coupled to spindles, a waxing and waning oscillation between 12 and 15 Hz generated by the thalamocortical system (Mölle et al., 2002; Steriade and Timofeev, 2003). On the other hand, functions associated with sleep slow waves were uncovered: they play a vital role in memory consolidation (Rasch and Born, 2013), synaptic plasticity (Tononi and Cirelli, 2014), and brain metabolic waste clearance by the recently discovered glymphatic system (Xie et al., 2013; Larsen et al., 2020). However, we do not know how these functions of sleep are dependent on slow-wave generation and their neuronal origin. This becomes even more problematic as research has shown that different types of slow waves exist: thalamocortical K-complexes vs. cortico-cortical slow waves (Steriade et al., 1993; Siclari et al., 2014). The different types of slow waves have different frequencies of occurrence in light and deep sleep; while large-amplitude steep-slope K-complexes are more prevalent in light NREM sleep (N2), the cortico-cortical waves occur more frequently as NREM sleep gets deeper (N3) (Genzel et al., 2014; Siclari et al., 2014; Halász, 2016; Bernardi et al., 2018). The spatial distribution of these two types of slow waves is different: K-complexes appear in the fronto-central area and are more focal as compared to cortico-cortical slow waves (Siclari et al., 2014). Interestingly, the two types of waves seem to be unequally reflecting recovery during sleep: no homeostatic change across the night in slow waves of largest amplitudes was found (Riedner et al., 2007). Moreover, it was shown that while there was a significant decline of SWA across the night for higher frequency slow waves (1.9–5 Hz), this was not the case for low-frequency slow waves (< 1.9 Hz) (Achermann and Borbély, 1997; Hubbard et al., 2020). As both the large amplitude and low frequency are characteristics of thalamocortical K-complexes, this subgroup of slow waves might not be associated with the recovery function of sleep.

Without a thorough understanding of how the different types of slow waves are driving the dissipation of sleep pressure and in turn are associated with sleep functions, it is impossible to establish a specific approach for promoting the restorative function of sleep. Specifically, do we have to boost cortico-cortical slow waves (and spare thalamo-cortical K-complexes) to promote recovery during sleep and how would we achieve this specificity? In this study, we demonstrate how PTAS allows boosting of physiological recovery during sleep and how this technique enables disentangling of the functional relevance of the different types of slow waves.

## Materials and Methods

### Participants

Data were obtained from 18 right-handed healthy young adults (23 ± 1.4 years old, nine females). All participants met the following inclusion/exclusion criteria: no personal or family history of neurological and sleep disorders, no severe brain injury, no known hearing deficits, no regular daytime napping, no current use of psychoactive agents or other medications, no traveling across more than 1 time zone in the 3 months before the study. Written informed consent was obtained prior to participation. The study was approved by the local ethics committee (Kantonale Ethikkommission Zürich, KEK-ZH) and performed according to the Declaration of Helsinki.

Out of 18 participants, four were excluded from the analysis because of poor sleep quality or a low number of presented stimuli. Out of the remaining 14 participants, three had low sleep efficiency and were excluded from correlation with behavioral readouts due to the incomparable sleep structure between the two experimental sessions. However, because the wake episodes occurred at the end of the night, it was possible to include all 14 participants in the analyses of the stimulation period and 13 participants in the analyses of the whole-night sleep dynamics. 3 out of 7 female participants included in the analyses of the stimulation period were taking oral contraceptives. For more details refer to the flowchart, Supplementary Figure 1.

### Experimental Protocol

Two experimental sessions in the sleep laboratory were separated by at least 1 week. Participants were instructed to keep a stable sleep-wake schedule for approximately 7 days (range from 4 to 8 days) prior to the experimental sessions in the laboratory. Compliance with the schedule was assessed using daily sleep diaries and wrist actigraphy (Actiwatch Type AWL from Cambridge Neurotechnology, CamNtech, Cambridge, United Kingdom). Participants did not take any medication at the time of the experiment and were required to keep caffeine consumption at their usual level and refrain from alcohol, high-intensity exercise, and sauna 24 h prior to each experimental session.

**FIGURE 1:**
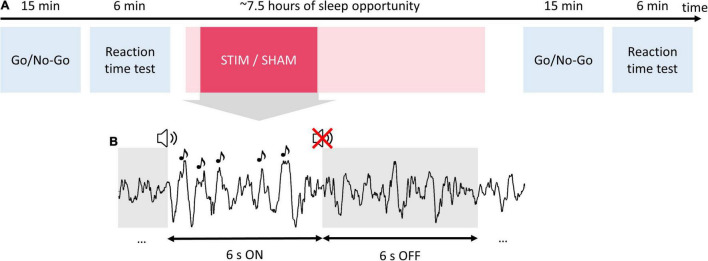
Study procedures and auditory stimulation protocol. Participants completed two experimental sessions in the sleep laboratory separated by 1 week. **(A)** During each experimental session, participants performed a Go/No-Go task and reaction time test in the evening before going to bed. Then participants were given a 7.5-h sleep opportunity. In one of the two experimental sessions, the phase-targeted auditory stimulation (stim) was applied for 2.5 h after the first stimulus onset. In the other session, no sounds were presented (sham). The next morning, at least 30 min after awakening, participants performed again the Go/No-Go task and reaction time test. **(B)** During the stimulation, pink noise pulses (1/f, 50 ms) were delivered in 6-s blocks (ON windows) followed by a 6-s pause (OFF windows).

The two experimental sessions in the lab had identical timelines (Figure 1A). Upon arrival, participants were prepared for the EEG measurement. Afterward, participants performed two behavioral tests (Go/No-Go task and reaction time test) approximately 1 h before lights off. Immediately prior to testing behavioral performance, participants estimated their subjective sleepiness on a slider scale “I can’t sleep right now” - > “This is the most I’ve ever wanted to sleep.” Bedtime was scheduled individually according to the participants’ preferences between 21:45 and 23:15, and sleep opportunity was approximately 7.5 h. In one of the two experimental sessions, the auditory stimulation targeting the ascending phase of slow waves (stim condition) was applied for 2.5 h, starting approximately 20 min from sleep onset, and during the rest of the night, no tones were presented. In the other session, no sounds were presented (sham condition), but time points of the possible stimulation were marked for offline analysis. The order of the sessions was randomized across subjects. Participants and experimenters were blind to the order of stim/sham conditions. The next morning, at least 30 min after awakening, participants estimated their subjective sleepiness and performed the Go/No-Go task and reaction time test again.

### Behavioral Measures of Attentional Performance

#### Go/No-Go Task

We used visual stimuli from a “whack-a-mole” version of the Go/No-Go task (Shapiro et al., 2014). This included three types of Go stimuli and 1 type of No-Go stimuli (3 to 1 Go/No-Go stimuli ratio was chosen to maximize false alarms in accordance with Young et al., 2018). The stimuli were presented for 300 ms with a variable inter-stimulus interval (ISI) from 550 to 1,200 ms. Prior to the main part of the task, participants were asked to complete a short training (30 trials, necessary correct response rate: 70%). The main part of the task was ∼15 min long (400 trials) and was separated into two blocks by a break of 1--3 min. The task was programmed using NBS Presentation software (Version 19.0, Neurobehavioral Systems, Inc., Berkeley, CA, United States^1^). To assess individual Go/No-Go task performance, we computed the number of errors (responses to No-Go trials) for both evening (E) and morning (M) sessions. As covariates for the correlation analysis with sleep variables, the normalized score was calculated as follows: M_stim/_E_stim_–M_sham/_E_sham_. This normalization procedure allowed us to assess the impact of stimulation on the behavioral measure while accounting for sleep-dependent performance gains. The resulting outcome variable is referred to as Go/No-Go Δ errors.

#### Reaction Time Test

We estimated the reaction time variability, one of the readouts of sustained attention sensitive to sleep pressure (Doran et al., 2001; Gunzelmann et al., 2009), in a simple reaction time test. We used the “Alertness” reaction time test as implemented in the Test of Attentional Performance (TAP) 2.3.1 software (Zimmermann and Fimm, 2002). In this test, reaction time is examined under two conditions to test phasic and tonic alertness: it includes a simple and cued reaction time task with a visual test stimulus (white cross on the black screen) and an auditory cue (four series of stimuli, two without cue stimulus, two with cue stimulus, ABBA design, 20 stimuli/series). For the correlation with sleep variables, we computed the standard deviation of RT across all trials, and normalized values according to the formula described above (TAP Δ std RT).

### High-Density Sleep Electroencephalography

Full-night sleep was recorded using high-density EEG (Net Amps 300 amplifier of the Geodesic EEG system (Electrical Geodesics, Inc.), Electrical Geodesics Sensor Net for long-term monitoring, 128 channels, referenced to a Cz electrode, sampling frequency 500 Hz). After adjusting the net to the nasion, vertex, and mastoids, all electrodes were filled with an electrolyte gel (ECI Electro-Gel, Electro-Cap International, Inc., Eaton, OH, United States). EEG electrode impedances were below 50 kΩ at the start of the recording and were re-checked in the morning. Electrooculography for sleep scoring was obtained by subtracting signals from the hd-EEG electrodes located above the eye and contralateral outer canthus of the eye. Submental electromyographic data were collected for visual sleep scoring using golden electrodes (Grass Technologies, West Warwick, RI, United States). Two additional golden electrodes were attached to the earlobes, which served as reference electrodes for manual sleep scoring. Impedances of the golden electrodes were below 10 kΩ.

#### Real-Time Phase-Locked Auditory Stimulation

In the stim condition, auditory stimulation was applied time-locked to the 35° target phase of slow waves detected in the Fp2 derivation (right prefrontal area). Real-time sleep classification, SWA detection, and EEG phase estimation were performed automatically by a configurable mobile EEG system (MHSL-SB; MHSL-SleepBand version 2, ETH Zürich, Switzerland) (Ferster et al., 2019). Stimulation triggers and triggers indicating borders of ON-OFF windows from the MHSL-SB were sent as a digital input to the EGI amplifier. For the MHSL-SB, three additional golden electrodes were placed on both mastoids and the right forehead, between electrodes 2 and 9 of the hd-EEG (the Fp2 location according to the standard 10–20 system). The latter was used for real-time EEG monitoring. The left mastoid electrode served as ground and the right mastoid electrode as reference. Impedances of the golden electrodes were below 10 kΩ. Auditory stimuli were delivered through soft headphones taped to the participant’s ears (SleepPhones, AcousticSheep LLC, Peninsula Drive, Pennsylvania, United States).

A detailed description of the MHSL-SB stimulation algorithm can be found in Ferster et al., 2019. In brief, a binary classifier categorized real-time acquired data as NREM or not-NREM sleep. This classification was performed by the estimation of delta- and beta-band power and their ratio from the prior 80 s. During NREM sleep, SWA detection, beta power increase detection, and EEG phase estimation based on a first-order phase-locked loop architecture were executed. Auditory stimuli (50 ms bursts of 1/f pink noise, approximately 50 dB SPL) were delivered through headphones whenever predefined SWA, EEG target phase, and beta power conditions were simultaneously met. The target phase was set at 35° after a positive-going zero-crossing. The minimal ISI was 500 ms. Stimuli were delivered in blocks of 6 s (ON windows) followed by a pause (OFF windows) of 6 s (Figure 1B). Note, that the beginning of the ON windows did not coincide with the presentation of the first stimulus and rather marked the beginning of the window of opportunity for stimuli to be presented. Stimulation was immediately stopped in case of arousal. Importantly, stimulation was applied for 2.5 h after the first stimulus, which was not earlier than 10 min of stable deep sleep.

### Electroencephalography Data Analysis and Statistics

Electroencephalography data were analyzed using Matlab R2017a and Matlab R2019b (Mathworks, Natick, MA, United States), with custom-written scripts and the FieldTrip toolbox (^2^ Oostenveld et al., 2011).

### Sleep Scoring and Artifact Rejection

For sleep scoring, the EEG data were consequently high-pass (FIR filter, 0.5 Hz) and low-pass filtered (FIR filter, 40 Hz), re-referenced to the earlobes, and downsampled to 128 Hz. Vigilance states (wake, N1, N2, N3, and REM) were visually scored by a sleep expert and verified by another sleep expert (author RH). Both of the scorers were blind to the experimental conditions. Scoring was performed using the signal from frontal, central, and occipital electrodes (20 s epochs) based on the American Academy of Sleep Medicine standard criteria (Berry et al., 2015). After sleep scoring, a semiautomatic procedure of artifact rejection and noisy channels identification was performed in 20 s epochs (Huber et al., 2000).

### Electroencephalography Preprocessing

Electroencephalography data were filtered between 0.5 (or 0.9 for participants with sweating artifacts, always the same for the two experimental nights) and 45 Hz using one-pass, zero-phase FIR filters (Hamming window, windowed-sinc FIR filter as implemented in the FieldTrip toolbox; lowpass: order 148, cutoff −6 dB at 45 Hz; highpass (*N* = 10): order 1,650, cutoff −6 dB at 0.5 Hz; highpass (*N* = 4 with sweating artifacts): order 918, cutoff −6 dB at 0.9 Hz). Data were downsampled to 250 Hz to speed up processing. 10 channels located on the earlobes and on the face below the front were excluded from further analysis. Noisy channels, identified by visual inspection, were interpolated using a spherical spline algorithm (4 ± 1 (mean ± std) channels were interpolated). The cleaned data were re-referenced to the average value across all 118 channels included in the analysis.

#### Response Analysis

Continuous data were segmented into 6-s epochs (ON-OFF windows pairs). To be included in the further analysis, at least 70% of each ON-OFF pair should have belonged to manually scored N2-N3 sleep stages. The ON-OFF pair should not have contained artifacts (identified in the previous step with semiautomatic artifact rejection), and the ON window should have had at least one slow-wave fulfilling criteria for stimulation (i.e., contained at least one stimulation trigger). An additional round of the semiautomatic artifact rejection was performed for the ON-OFF pairs (11 ± 7 windows were excluded per one recording). The final number of windows included in the analysis did not differ between the two conditions (paired, *t*_13_ = −1.54, *p* = 0.15, stim: 286 ± 76, sham: 321 ± 65).

#### Homeostasis Analysis

Continuous data were segmented into 20-s epochs, corresponding to sleep stages. Only artifact-free epochs and sleep stages N2 and N3 were included in the analysis.

### Frequency Analysis

#### Spectral Analysis

To estimate the EEG power in different frequency bands, we used Welch’s power spectral density estimate (PSD, *pwelch* function as implemented in Matlab). Slow-wave activity (SWA; power in the 1–4 Hz range) was determined for 6-s ON/OFF windows for response analysis and 20-s sleep stage epochs for homeostasis analysis, as numerical integration of power in the defined frequency interval.

**FIGURE 2:**
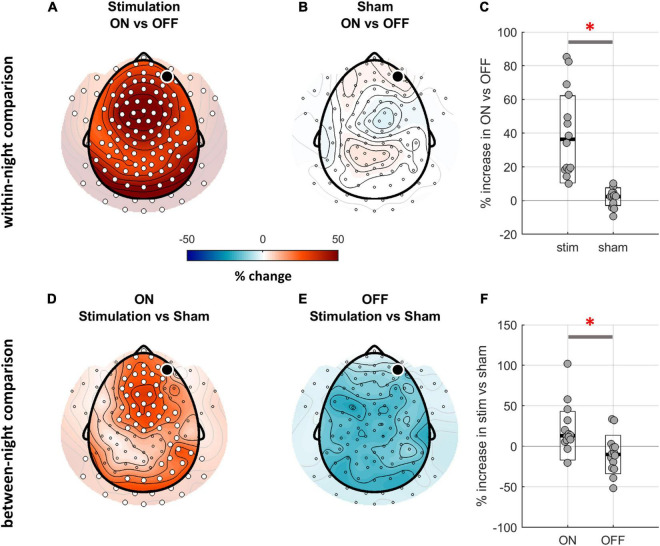
Comparison of SWA (1–4 Hz) between ON and OFF windows of stim and sham nights. **(A,B)** Topographical distribution of SWA for the within-night comparison of ON and OFF windows. The black dot indicates the target channel Fp2, white dots indicate a significant change. While the ON-OFF-window contrast for the stim night reveals a global SWA increase in ON windows (cluster corrected paired two-sided *t*-test, *N* = 14, *p*_clust_ < 0.001, *d* = 1.18), the same contrast for the sham night does not show any differences. **(C)** The relative SWA change between ON and OFF windows across all electrodes was significantly larger for the stim night as compared to sham night (average across all channels, *t*_13_ = 5.07, *p* < 0.001; black line indicates median, white box with black outline indicates CI, red asterisk indicates significance). **(D,E)** Topographical distribution of SWA for the between-night comparison of ON and OFF windows. The contrast of ON windows between stim and sham nights confirms a stimulation-related boost of SWA (*N* = 14, *p*_clust_ = 0.01, *d* = 0.81). **(F)** The average increase in SWA when contrasting stim and sham night was significantly higher for the ON windows as compared to OFF (average across all channels, *t*_13_ = 4.55, *p* = 0.001).

**FIGURE 3:**
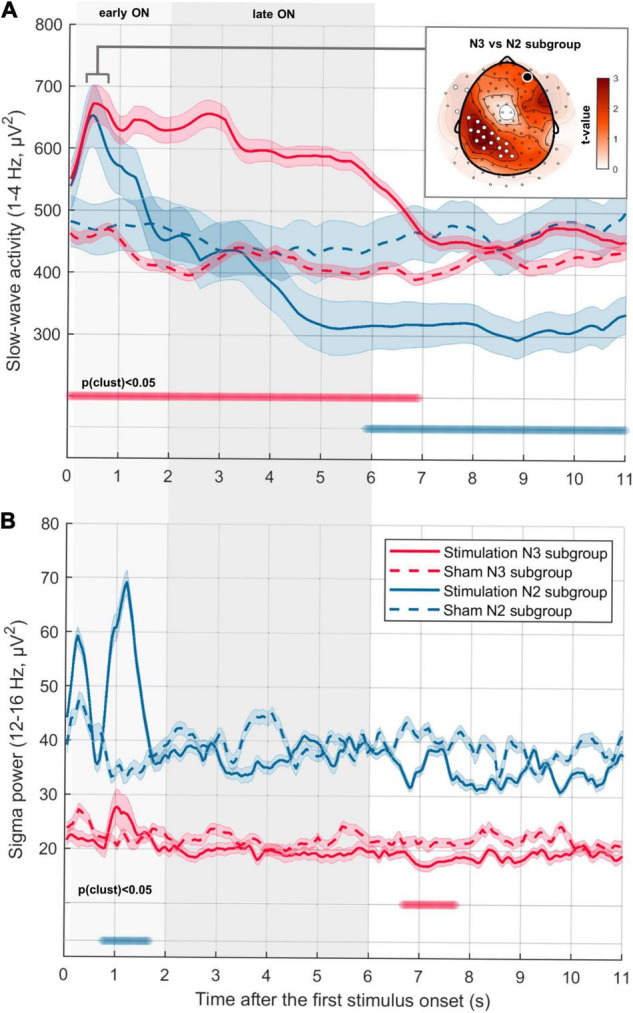
Temporal dynamics of SWA and sigma power depending on sleep depth. **(A)** Time-resolved dynamics of SWA (1–4 Hz) in consecutive ON-OFF windows for participants with a high percentage of N3 (red line, “N3 subgroup,” *N* = 7) and participants with a high percentage of N2 (blue line, “N2 subgroup,” *N* = 7) during the stimulation period (first 2.5 h from sleep onset). Median ± CI (indicated by shaded area around lines) power averaged across all channels is aligned to the first stimulus in ON-windows (first stimulus onset in the Stimulation condition for N3 subgroup: 0.96 ± 0.14 s; for N2 subgroup: 1.26 ± 0.23 s). Thus, the OFF window starts on average after 5.04 s in the N3 subgroup and 4.74 s in N2 subgroup. First 6 s were divided into earlyON (0.2–2 s) and lateON (2–6 s) to account for the potential presence of a K-complex evoked by the first stimulus. Thick horizontal bars indicate time points of significant difference between conditions (cluster corrected paired two-sided *t*-test, N3 subgroup: *p*_clust_ < 0.001, *d* = 1.31; N2 subgroup: *p*_clust_ < 0.001, *d* = –1.18). The inserted figure represents the topography of the difference in SWA change in N2 and N3 subgroups during the first induced negative peak (0.3–0.8 s after the first stimulus onset). The black dot indicates the target channel Fp2, white dots indicate a significant increase (cluster corrected two-sided unpaired *t*-test, *N* = 7, *p*_clust_ = 0.03, *d* = 1.67). **(B)** Time-resolved dynamics of sigma activity (12–16 Hz) in consecutive ON-OFF windows (same as A) (cluster corrected paired two-sided *t*-test, N3 subgroup: *p*_clust_ = 0.02, *d* = –1.68; N2 subgroup: *p*_clust_ = 0.01, *d* = 2.64).

**FIGURE 4:**
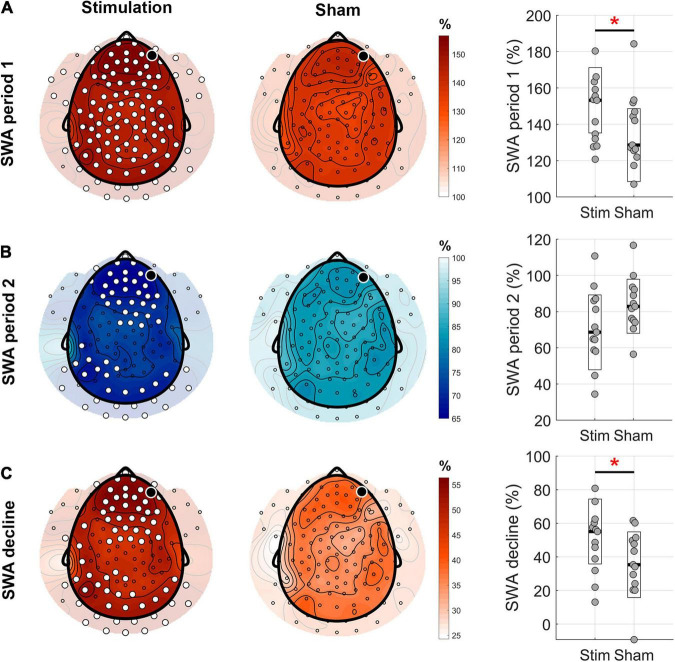
Topographical distribution of the homeostatic response to the phase-targeted auditory stimulation. **(A)** Mean slow-wave activity (SWA) in the first 2.5 h period from sleep onset (period 1) normalized by mean SWA during the entire night in the stimulation and sham conditions (white dots indicate significant changes between the two conditions, cluster corrected paired two-sided *t*-test, *N* = 13, *p*_clust_ = 0.004, *d* = 0.97). Boxplots (right hand column) show a comparison of normalized values averaged across all channels for stim and sham conditions (*t*_12_ = 3.26, *p* = 0.007, red asterisk indicates significant change). **(B)** Mean SWA in the following 2.5 h period (period 2) normalized by mean SWA during the entire night, in stimulation and no stimulation conditions (cluster corrected paired two-sided *t*-test, *N* = 13, *p*_clust_ < 0.024, *d* < –0.51). On average, across all channels, there was a trend toward lower SWA in sham versus stim (*t*_12_ = –2.12, *p* = 0.055). **(C)** SWA decline in percent from the first 2.5 h to the following 2.5 h of sleep showed a rather global, stronger decline in the stimulation compared to the sham condition (cluster corrected paired two-sided *t*-test, *N* = 13, *p*_clust_ < 0.023, *d* > 0.51). On average across all channels, the SWA decline was higher in stim compared to sham condition (*t*_12_ = 2.37, *p* = 0.035, red asterisk indicates significance). The black dots indicate the target channel Fp2.

For the response analysis, power was estimated in 6-s ON/OFF windows [FFT, Tukey window (*r* = 0.5), average of two 4-s epochs overlapping by 2 s], resulting in a 0.25 Hz frequency resolution, and then averaged for ON and OFF windows and for each condition and participant separately. These measures were used for the topographical representations of SWA change (Figures 2–4). For the power spectral density plots, the estimates were additionally transformed to dB [10 × log10(μV^2^/Hz), Supplementary Figures 2, 4].

For the homeostasis analysis, spectral analysis of EEG channels was performed on consecutive 20-s sleep stage epochs [FFT, Tukey window (*r* = 0.5), average of nine 4-s epochs overlapping by 2 s], resulting in a 0.25 Hz frequency resolution. To investigate the dynamics of sleep homeostasis and the effect of PTAS on SWA decline across the night, we assessed the stimulation effect in NREM sleep (stages 2 and 3) in the 2.5 h after sleep onset (period 1) and the post-stimulation effect in the following 2.5 h (period 2). To account for individual differences, as well as night to night variability in the same participant, mean SWA in period 1 and period 2 were normalized by mean SWA across the entire night, resulting in a relative change from the mean overnight SWA. The homeostatic decline in sleep pressure across the night was calculated as a decline in mean SWA from period 1 to period 2 (1–SWA2/SWA1). It was not possible to accurately estimate SWA decline in case of fragmented sleep occurring in the first cycle. Therefore, in this case, SWA in the fragmented period was replaced by the SWA from the following NREM epochs (1 subject, approximately 40 min).

#### Time-Resolved Frequency Analysis

Prior to performing the time-frequency transform, adjacent ON-OFF windows (6 s each) were appended, resulting in one 12-s long epoch. Time-resolved power was calculated by using wavelets with a Hanning taper and an adaptive time window for each frequency (*ft_freqanalysis* function (“mtmconvol” method) as implemented in FieldTrip toolbox). Power was estimated in the delta band between 1 and 4 Hz (4 full cycles per window, ΔT = 4/f) for slow-wave activity, and from 12 to 16 Hz (6 full cycles per window, for 12–16 Hz) for sigma activity in steps of 0.04 s and a frequency resolution of 0.25 Hz. Power was calculated for each epoch separately. Before averaging the resulting envelope of power across epochs, each epoch was re-aligned to the position of the first stimulus. After this, the epochs were averaged for each condition and subject separately.

### Statistical Analysis

No statistical methods were used to predetermine sample size, however, it was similar to those of previously reported studies, where sleep was assessed using high-density EEG recordings (Huber et al., 2004). Statistical analyses were carried out in Matlab R2017a and R2019b, and IBM SPSS, version 27. Statistical significance level (alpha) was set to 0.05 for all tests. To evaluate the influence of PTAS on the immediate SWA change (response analysis) and overnight SWA decline (homeostasis analysis), we conducted repeated-measures analysis of variance (rmANOVA). For the Response analysis, we included the following within-subject factors: condition (stim vs sham) and window (ON vs OFF). For the Homeostasis analysis, we included the following within-subject factors: condition (stim vs sham) and period (first 2.5 h and the following 2.5 h). Normalized SWA variables were log_10_ transformed prior to statistical analysis. To compare variables of interest or perform *post hoc* tests, we used 2-tailed *t*-tests (paired for dependent samples and unpaired for independent samples). *Post hoc t*-tests were performed either on the average across all channels or at the topography level. Pearson’s correlation coefficient was computed to assess the association between two variables of interest both for the average across all channels and at the topography level.

To correct for multiple comparisons when performing *t*-tests or correlation on the topography level, we used a non-parametric clustering procedure (Maris and Oostenveld, 2007; Maris, 2012). First, paired or unpaired *t*-test for the contrast of interest or Pearson correlation was performed separately for all electrodes. Next, significant neighboring electrodes were clustered if they showed the same direction of effect. To assess the statistical significance of each cluster, a cluster-level test statistic was calculated by computing the sum of all t-values in the cluster (for correlations after an additional step of converting R-values to t-values). The significance of each cluster was estimated by comparing the cluster-level test statistic to a reference permutation distribution derived from the data. The reference distribution was obtained by randomly permuting the data 5,000 times. The cluster p-value was estimated as the proportion of the elements in the reference distribution exceeding the cluster-level test statistic. An analogous procedure was used for the cluster correction of one-dimensional measures like power spectral density plot (cluster was formed from the adjacent frequency bins) or time-resolved output of wavelet transform (cluster was formed from the adjacent time points). Cohen’s d of the cluster (for paired or unpaired samples) was assessed by using a corresponding formula (Cohen, 1988) on the average across significant cluster units. The effect size can be interpreted as follows: 0.2 = small effect, 0.5 = moderate effect, 0.8 = large effect.

## Results

### Sleep Macrostructure

In a first step, we assessed stimulation-induced changes in sleep macrostructure based on the visually scored sleep stages (Supplementary Table 1). We found no differences between the two conditions, except for a lower percent of sleep spent in NREM sleep and higher percent in REM sleep in the stimulation condition. When comparing the amount of NREM sleep (N2 and N3) during our main analysis interval (period 1 and period 2) we found no significant differences between stim and sham conditions.

### Topographical Analysis of the Response to the Phase-Targeted Auditory Stimulation

In a second step, we characterized the spatial properties of the response to phase-targeted auditory stimulation. To test whether PTAS influenced SWA during the stimulation period (averaged across all electrodes), we computed a repeated-measures ANOVA using *window* (ON, OFF) and *condition* (stim, sham) as within-subject factors. This analysis yielded a significant main effect of *window* (*F*_1,13_ = 24.6, *p* < 0.001) and interaction of the two factors (*F*_1,13_ = 16.6, *p* = 0.001). To further elucidate the origin of these effects, we performed *post hoc t*-tests. To begin with, we investigated the within-night stimulation effects by comparing ON and OFF windows for stim (stimON and stimOFF) and sham (shamON and shamOFF) for each electrode separately. PTAS led to an increase in SWA (1–4 Hz) in a cluster spanning the entire cortex (*p*_clust_ < 0.001, Figure 2A). As expected from a homeostatic response, this power difference between ON and OFF windows was most pronounced in the low frequency range but also included changes in other frequencies (Supplementary Figure 2).

One limiting factor of this within-night comparison is that the observed increase in SWA could be explained either by the stimulation-related increase in ON windows or by a decrease in OFF windows. To test these two possibilities, in a second step, we performed a between-night (stim vs sham) comparison of ON and OFF windows. The between-night contrast of ON windows (stimON vs shamON) revealed a bilaterally symmetric SWA increase over fronto-central and occipital areas and a unilateral increase over the right parietal area (*p*_clust_ = 0.01, Figure 2D). Therefore, auditory stimulation resulted in a SWA increase, irrespective of whether assessed by contrasting ON/OFF windows within one night (Figure 2C), or ON windows of stim and sham nights (Figure 2F). The topographical analysis also revealed a trend for a decrease in SWA when comparing stimOFF to shamOFF, however, this contrast did not survive cluster correction (p_clust_ = 0.06, Figure 2E). Thus, the decrease in OFF windows might contribute to the overall difference between ON and OFF windows in within-night contrast. Following this notion, we focused on between-night contrasts (stimON vs shamON and stimOFF vs shamOFF) in subsequent analyses.

To exclude that the stimulation effect when comparing stim and sham is affected by a selection bias (i.e., preferential start of ON-windows in the period of high SWA during stim condition), we tested whether the slow-wave detection algorithm performed equally well in stim and sham conditions. To do so, we compared the number of stimuli (in case of the sham night, the time points of the possible stimuli) and ON-window properties. The number of stimuli did not differ between the stim and sham conditions [stim night: 1,053 ± 329 (mean ± std), sham: 1,245 ± 300, *p* = 0.65]. The mean number of stimuli per ON window (stim: 4.6 ± 0.5, sham: 4.3 ± 0.5, *p* = 0.07) and the proportion of ON windows belonging to sleep stage N3 (stim: 56 ± 27%, sham: 50 ± 20%, *p* = 0.64) were also similar for stim and sham conditions. Hence, PTAS did not cause a change in the number of waves fulfilling the criteria for stimulation but rather affected the overall power in the SWA range.

Figures 2C,F show high variability of the change in SWA across participants. This variability might be related to a dose-dependent effect of PTAS on SWA, i.e., the more stimulations, the larger the effect. In fact, despite the high replicability of the percent of ON windows belonging to the N3 stage across the two experimental conditions (*R* = 0.69, *p* = 0.006), there were pronounced inter-individual differences in the proportion of N3 (range from 5 to 89% of NREM sleep). In other words, some participants slept deeper than others irrespective of the experimental condition. Furthermore, as expected by the definition of N3 as a sleep stage enriched with slow waves, a high proportion of N3-ON windows was associated with a high number of stimuli per window (*R* = 0.56, *p* = 0.04) and a high total number of stimuli (*R* = 0.62, *p* = 0.02).

Taking into account these findings, we then addressed the question of whether the SWA change observed in the between-night contrasts is influenced by sleep depth during the stimulation period. To do so, we performed a correlation analysis of the relative SWA change (stim vs sham) in ON and OFF windows and the percentage of N3-ON windows. The positive correlation between the SWA change in ON windows and the percentage of N3-ON windows was rather global (Supplementary Figures 3A,B, *R* = 0.65, *p* < 0.001), but spared the fronto-central area. Similarly, the change in SWA seen in OFF windows also globally positively correlated with the percentage of N3 windows (Supplementary Figures 3C,D, *R* = 0.72, *p* < 0.001). Thus, a higher percentage of N3 windows during stimulation was related to a larger SWA increase in ON windows and a less pronounced SWA decrease in OFF windows (Supplementary Figures 3B,D).

### Temporal Evolution of the Response to the Phase-Targeted Auditory Stimulation: Different Properties of Slow Waves Evoked by Auditory Stimulation Applied During N2 and N3 Sleep

To characterize the temporal evolution of stimulation-evoked power changes we used a wavelet time-frequency decomposition. Given the strong association between the effect of PTAS on SWA and the proportion of N3 during the stimulation period, we performed a median split of participants into an N3 (>50% of ON windows belong to N3, *N* = 7) and N2 (<50% of ON windows belong to N3, *N* = 7) subgroups. Of note, the mean number of stimuli per ON window was not significantly different for the two subgroups (*p* = 0.16).

Slow-wave activity response had a different temporal development in the two subgroups (Figure 3A). For this analysis, we aligned the consecutive ON-OFF windows to the first stimulus onset. While for the N3 subgroup auditory stimulation resulted in a plateau-like SWA increase lasting for 6 s (earlyON and lateON, Figure 3A, *p*_clust_ < 0.001), the SWA increase in the N2 subgroup was rather transient (< 2 s, earlyON, Figure 3A) and did not survive cluster correction. Another subgroup difference concerns the time > 6 s after the first stimulus onset, where SWA in the N3 subgroup returned to a level comparable to sham. In contrast, SWA in the N2 subgroup decreased below the sham level and remained low (Figure 3A, *p*_clust_ < 0.001). As K-complexes have a specific spatial representation, we next investigated whether spatial properties of the evoked slow waves might differ depending on sleep depth. To do so, we compared the SWA topography of the two subgroups during the time interval of the first evoked peak of SWA (Figure 3A, inserted). The resulting topography showed that the first stimulation-evoked negative peak had a broader topographical distribution in the N3 subgroup as compared to the N2 subgroup, showing a ring-shaped cluster of electrodes with a significant difference (Figure 3A, inserted, *p*_clust_ = 0.03). In addition to SWA, we also assessed the time-course of sigma band (12–16 Hz) power including the frequency range of spindles. Evoked K-complexes are often described as a multicomponent phenomenon, including the bi-phasic time-locked delta wave and spindles associated with the induced up phase (Amzica and Steriade, 1997). Thus, an increase in sigma power at around 1 s after the first stimulus can provide indirect evidence for an evoked K-complex. And indeed, the time-course analysis of sigma power change demonstrated a stimulation-evoked sigma power enhancement in the N2 subgroup peaking around 1 s after the first stimulus onset (Figure 3B, *p*_clust_ = 0.009).

To sum up, these analyses indicate different components of the effect of PTAS on SWA that nicely separates the N2 and N3 subgroups. Participants of the N3 subgroup demonstrated a stable stimulation-driven increase in SWA lasting the entire duration of the ON window (without an associated increase in sigma power) and no negative rebound after the end of the ON window. In contrast, participants of the N2 subgroup showed a transient SWA increase and a simultaneous increase in sigma power, which altogether resembles the pattern of an evoked K-complex.

### Homeostatic Response to Phase-Targeted Auditory Stimulation Assessed by Means of Slow-Wave Activity and Slow-Wave Activity Decline Across the Night

After having established the immediate effects of PTAS on SWA in ON and OFF windows separately, we were interested in more global effects of overnight SWA including both ON and OFF windows, in particular whether PTAS induces homeostatically regulated sleep. In order to achieve this, we calculated SWA in the first 2.5 h (period 1) and in the following 2.5 h of sleep (period 2), and the decline of SWA from period 1 to period 2. As one subject had poor sleep efficiency during the second part of the night, it was excluded from further analyses (Supplementary Figure 1). To account for intra- and inter-individual differences across subjects, SWA for each of the periods was normalized by the average SWA across the night. A repeated-measures ANOVA, assessing the influence of condition (stim and sham) and period (period 1 and period 2) on SWA, showed a significant main effect of *period* (*F*_1,12_ = 51.01, *p* < 0.001) and a significant interaction *condition* × *period* (*F*_1,12_ = 7.5, *p* = 0.02). SWA was higher in the stim compared to the sham condition during the first 2.5 h on average across all electrodes (Figure 4, right column, *p* = 0.007). In the following 2.5 h, SWA tended to be lower in the stim compared to the sham condition (*p* = 0.055). *Post hoc* tests for topographical differences were further conducted between conditions for each period. Consistent with our observations for the between-night ON-window comparison, this analysis revealed a global increase in SWA in period 1 in the stimulation compared to the sham condition in almost all channels (*p*_clust_ < 0.01, Figure 4A). In period 2, SWA was decreased in the stimulation compared to sham condition, primarily over frontal and occipital areas (*p*_clust_ = 0.02, Figure 4B). This lower SWA in the second period of sleep may indicate a rebound effect after the stimulation. Next, we investigated the homeostatic decline of SWA across the night and calculated the percentage change of SWA from period 1 to period 2 (1–period2/period1). A stronger decline of SWA in the stim compared to the sham condition is present globally, i.e., across most electrodes (*p*_clust_ = 0.02, Figure 4C). This result indicates faster dynamics of sleep homeostasis (i.e., faster dissipation of sleep pressure) due to PTAS during the first 2.5 h of sleep.

In order to eliminate the influence of night-to-night differences in sleep stages, the SWA decline in the stim condition was normalized to sham for further analyses. To investigate a more direct relationship between the SWA decline and the stimulation, we performed a correlation analysis between the number of stimulations and the average SWA decline across all channels normalized to sham. This analysis showed a positive correlation between the SWA decline and the total number of stimulations (*R* = 0.69, *p* = 0.02), as well as the mean number of stimulations per ON window (*R* = 0.65, *p* = 0.03). These results indicate that PTAS is able to boost the homeostatic decline of SWA across the night in a dose-dependent manner.

### Relationship Between the Slow-Wave Activity Decline and Slow-Wave Characteristics During the Stimulation Period

We evaluated which electrophysiological features during the stimulation period contributed most to the acceleration of the homeostatic decline in SWA across the night. To do so, we performed correlation analyses between the SWA decline and the electrophysiological features of the stimulation response, i.e., the earlyON SWA response (averaged power across 0.1–2 s after the first stimulus onset), the earlyON sigma power response (0.7–1.5 s after the first stimulus), the lateON SWA response (2–6 s after the first stimulus), and the SWA during the OFF window (6–10 s after the first stimulus). All values were computed as a ratio of the stim and sham conditions after averaging across all channels. To capture the pronounced change in SWA and sigma power between the earlyON and lateON (which might indicate the presence of K-complex), the earlyON response was expressed relative to the lateON response in the corresponding frequency band. Without this procedure of normalization, earlyON would reflect the overall level of SWA at the beginning of the ON windows and not a change of power between the earlyON and lateON. The above-mentioned time windows for the electrophysiological features were selected based on the significant clusters in the wavelet analysis (see Figure 3). The SWA decline was positively correlated with the lateON SWA response (*R* = 0.66, *p* = 0.03), and SWA during the OFF-window (*R* = 0.67, *p* = 0.02). However, there were no significant correlations between neither the SWA decline and earlyON SWA response, nor between the SWA decline and the earlyON sigma response. These results indicate that the boosted SWA decline is driven by the lateON SWA response and not by the early, K-complex-like response.

As there was a significant change in percent spent in NREM and REM sleep between the two conditions, we further assessed whether the observed changes in SWA decline and slow-wave characteristics are driven by the change in sleep macrostructure. However, there were no significant correlations between the SWA change during the stimulation, SWA decline, and percentage spent in NREM or REM sleep (*R* < 0.12, *p* > 0.74).

### Association of Day-Time Behavioral Performance With the Response to Stimulation and the Homeostatic Decline in Slow-Wave Activity

During each night session, before and after sleep, participants performed two behavioral tests (Methods, Figure 1): a visual Go/No-Go and a simple reaction time test (“Alertness” in the TAP test battery). We included in our analysis the number of errors reflecting inhibitory control failures in the Go/No-Go task and the standard deviation of reaction time (std RT) as a readout of arousal and sustained attention in the simple reaction time test (Weissman et al., 2006). In addition to the measures of attentional performance, we collected assessments of subjective sleepiness prior to each session of behavioral tests.

Out of 11 participants included in the behavioral performance analysis (see Supplementary Figure 1), due to technical issues, only eight had estimates of subjective sleepiness. An exploratory analysis performed in these eight participants showed no significant difference between the two sessions (*p* = 0.1).

When analyzing the outcome of behavioral tasks, first, we compared Go/No-Go errors and std RT between the experimental conditions. To assess overnight changes, we divided the values of the morning session by the values of the evening session. There was no significant difference in the overnight change of Go/No-Go errors (*p* = 0.6) between the stim and sham sessions. The overnight change in std RT was also not significantly different (*p* = 0.9) between the conditions.

In a second step, we correlated the behavioral measures and features of the response to stimulation derived from the wavelet analysis. To account for the sleep-dependent improvement in behavioral performance, we normalized overnight changes in the stim condition by the sham condition (Go/No-Go Δ errors and TAP Δ std RT). We found that the early response, both in the SWA and sigma frequency band, was associated with more Go/No-Go Δ errors (SWA: *R* = 0.69, *p* = 0.02; sigma: *R* = 0.72, *p* = 0.01). No association with TAP Δ std RT was found. Conversely, the SWA increase during the lateON SWA response and the OFF window was negatively correlated with TAP Δ std RT (late response: *R* = −0.72, *p* = 0.01; OFF-window: *R* = −0.67, *p* = 0.02). No association with Go/No-Go Δ errors was found. In summary, the earlyON SWA response is associated with worse performance in the Go/No-Go task the next morning whereas the boost in SWA in the second part of the ON window (lateON SWA response) is related to a decrease in reaction time variability in the TAP task.

In a final step, behavioral measures were related to our marker of the homeostatic decline of sleep pressure across the night. The SWA decline was negatively correlated with TAP Δ std RT (*R* = −0.65, *p* = 0.03), indicating an association between the boosted SWA decline and decreased reaction time variability the following morning. No associations with the Δ errors in the Go/No-Go task were observed.

## Discussion

Our results suggest that PTAS enhances sleep in a physiological way, i.e., as predicted by the generally accepted concept of homeostatic sleep regulation (Achermann and Borbély, 2003). This concept predicts that (i) a positive rebound after the stimulation period would imply that recovery during stimulated sleep had been disrupted, (ii) no rebound after the stimulation period would imply that the stimulation did not affect recovery, and (iii) a negative rebound would indicate that recovery during stimulated sleep was boosted. Moreover, according to this concept, the changes would be most pronounced in the SWA frequency range. The latter two points are exactly what we found: We observed a negative rebound after the stimulation period, which was best visible in the SWA frequency range (see Supplementary Figure 4).

Importantly, overall sleep macrostructure remained largely intact, with higher percent of sleep spent in NREMS and lower in REMS in the stim condition. However, the difference was small (2% in both cases) and did not correlate with to our primary outcome measures, i.e., the SWA boost during the stimulation and the decline of SWA across the night. Hence, PTAS seems to facilitate the dissipation of homeostatic sleep pressure leaving the overall sleep structure largely intact.

Interestingly, and again as expected from a boost in physiological sleep, a faster dissipation of the homeostatic sleep pressure was associated with an improved cognitive performance the next day. These results further support PTAS as a non-invasive approach to enhance the recovery function of sleep, which might be beneficial for various populations suffering from insufficient sleep recovery (Rothman and Mattson, 2012). Future studies need to prove that these effects can be replicated in a home setting over prolonged periods. Wearable technology allowing for PTAS (Ferster et al., 2019; Arnal et al., 2020; Grifantini, 2020) renders such trials feasible.

Besides this promise for sleep therapy, our important result is that the PTAS-mediated SWA boost can be divided into two distinct components—an early and late response. The early response, immediately after the first stimulus, was most pronounced in light sleepers and resembled an evoked K-complex. Not only was this initial response associated with increased sigma power, but it also had a distinct frontal hotspot topography, both features indicative of an evoked K-complex (Cote et al., 1999). The late response, which was prominent in deep sleep, had a broader fronto-central distribution, was not associated with a sigma increase, and lasted as long as the 6-s ON window. Notably, depending on sleep depth, the PTAS-mediated effect on SWA was also different during the OFF windows: in contrast to a deep sleep where SWA during the OFF windows returned back to sham levels, in light sleep the subsequent OFF windows were characterized by a decrease of SWA as compared to sham. This observation might be interpreted either as a sign of diminished slow-wave generation following K-complex-like activity in ON windows (Halász et al., 2004). Alternatively, it might be related to the redistribution of sporadic K-complexes, typical for light sleep, toward ON windows as a consequence of the refractory period after the evoked K-complex (Mak-McCully et al., 2014). Based on these findings, we propose that PTAS, depending on the sleep depth, has a distinct effect on slow-waves: While PTAS during light sleep more frequently elicits K-complex-like waves, during deep sleep it results in successful boosting of SWA throughout the ON window (Siclari et al., 2014; Bernardi et al., 2018; Kim et al., 2019).

The separation into an early and late component is of high relevance as they seem to contribute differently to sleep-dependent recovery. The boost in SWA decline was associated with the late component, i.e., the sustained increase in SWA primarily found during deep sleep. In contrast, the early, K-complex-like response was not associated with a boost in SWA decline. Hence, not all slow waves might contribute equally to recovery during sleep. This conclusion is in agreement with the previous findings showing that different types of slow waves exist (Siclari et al., 2014; Bernardi et al., 2018) and that subtypes of slow waves (i.e., with large amplitude and low frequency (Achermann and Borbély, 1997; Riedner et al., 2007) do not show a homeostatic decline across the night. Strikingly, the two SWA components not only had distinct effects on SWA decline but also related to specific behavioral consequences. We found that the late response, linked to a boosted homeostatic decline, was associated with an improvement in sustained attention, as reflected in less variable reaction times in the simple reaction time test. Conversely, if the early response was pronounced, which was associated with a slower homeostatic decline, participants showed decreased inhibitory control, as reflected in an increase of committed errors during the Go/No-Go task the next morning. To recapitulate, when applied during deep sleep, PTAS seems to facilitate recovery and improve sustained attention. When applied during light sleep, PTAS did not have such an effect. Thus, to maximize the boost in recovery during sleep, we propose to limit PTAS to deep sleep.

In summary, we show that PTAS is a promising approach to boost recovery during sleep. From a basic research perspective, our results support the notion that different types of slow waves exist, and that they might not equally contribute to recovery during sleep. It would be interesting to know which type of slow waves is associated with the proposed functions of sleep, from synaptic homeostasis to glymphatic clearance. This, in turn, would help to improve PTAS for patients with impaired sleep recovery associated with various neurological and psychiatric disorders (Rothman and Mattson, 2012).

### Limitations of the Current Approach and Outlook

The main limitation of this study is a rather small sample size, which did not allow a complex statistical analysis that takes into account parameters such as sex, age, estrous cycle, etc. Moreover, we would also like to note that the observed correlations between SWA change during the stimulation, SWA decline across the night, and behavioral readouts do not allow to draw any causal conclusions. Similarly, we cannot conclude if a negative rebound in the second half of the night after boosting SWA in the first part of the night is essential for the faster dissipation of sleep pressure and improved morning performance. To address these research questions, one might use within-subject study designs and more elaborated stimulation algorithms targeting different types of slow waves.

Following the recent discussion on the distinct contribution of low (1–2 Hz) and high (3–4 Hz) SWA in the homeostatic SWA decline (Hubbard et al., 2020), it might be of interest to address how slow waves with different periods and slopes might be affected by PTAS. Finally, future studies are needed for a more precise definition of different types of slow waves, as there are multiple classifications that may or may not reflect the same phenomenon (Terzano and Parrino, 2000; Steriade and Timofeev, 2003; Siclari et al., 2014).

## Data Availability Statement

The datasets presented in this article are not readily available because the ethical approval granted to the authors by the IRB does not allow the publication of the raw data online. If readers would like to re-analyze the dataset (for different purposes), additional ethical approval (on an individual user and purpose basis) will be required. The authors would be happy to support additional ethical approval applications from researchers for access to this dataset. The datasets generated for this study are available upon reasonable request to the corresponding author. Requests to access the datasets should be directed to RH, reto.huber@kispi.uzh.ch.

## Ethics Statement

The studies involving human participants were reviewed and approved by Kantonale Ethikkommission Zürich, KEK-ZH. The patients/participants provided their written informed consent to participate in this study.

## Author Contributions

RH, EK, and JS designed the study. EK and JS recruited the participants, collected the data, and analyzed the data. SL and SS helped with the data collection and sleep scoring. GS, MF, and WK contributed to the data analysis. MF, GP, and WK developed the phase-targeted auditory stimulation device used in the study and offered their support and expertise. EK, JS, and RH wrote the manuscript. All authors commented on the manuscript, proposed corrections, and agreed on the final version of the manuscript.

## Conflict of Interest

RH and WK are shareholders of Tosoo AG, a company developing wearables for sleep electrophysiology monitoring and stimulation. Tosoo AG did not contribute in any form to the work presented in this manuscript. The remaining authors declare that the research was conducted in the absence of any commercial or financial relationships that could be construed as a potential conflict of interest.

## Publisher’s Note

All claims expressed in this article are solely those of the authors and do not necessarily represent those of their affiliated organizations, or those of the publisher, the editors and the reviewers. Any product that may be evaluated in this article, or claim that may be made by its manufacturer, is not guaranteed or endorsed by the publisher.

## Footnotes

1
www.neurobs.com

2
http://fieldtrip.fcdonders.nl/
