# Supplementary material for: Boosting Recovery During Sleep by Means of Auditory Stimulation
Source: Front Neurosci. 2022 Feb 2;16:755958. doi: 10.3389/fnins.2022.755958 (PMC8847378; doi:10.3389/fnins.2022.755958)
Supplement: Supplementary file 1 [file Table_1.DOCX]

Supplementary Material

# Supplementary Tables

**Supplementary Table 1.** **Sleep macrostructure.**

Sleep parameters were calculated from the visual scoring of sleep stages (N=14). Sleep efficiency was calculated by dividing total sleep time with total time in bed. Wake after sleep onset is expressed as the percentage of total time in bed. Sleep stages (NREM sleep, stage N1, stage N2, stage N3, and REM sleep) are expressed as a percentage of total sleep time. P-values result from paired Student’s t-tests. Bold letters indicate a significant difference (p<0.05). Sleep macrostructure did not differ between the two nights except for increased proportion of REM sleep at the expense of decreased NREM proportion.

|  | stim | | sham | |  |
| --- | --- | --- | --- | --- | --- |
|  | mean | std | mean | std | p-value |
| Sleep parameter |  |  |  |  |  |
| Total time in bed [h] | 7.6 | 0.17 | 7.6 | 0.16 | 0.59 |
| Total sleep time [h] | 6.8 | 0.71 | 6.6 | 0.78 | 0.34 |
| Sleep efficiency [%] | 90 | 10 | 86 | 10 | 0.38 |
| Sleep latency [min] | 19 | 10 | 26 | 14 | 0.1 |
| Wake after sleep onset [%] | 6 | 7 | 8 | 8 | 0.36 |
| NREM sleep [%] | 79 | 3 | 81 | 5 | **0.03*** |
| Stage N1 [%] | 5 | 2 | 5 | 1 | 0.93 |
| Stage N2 [%] | 56 | 10 | 56 | 12 | 0.71 |
| Stage N3 [%] | 18 | 10 | 20 | 12 | 0.24 |
| REM sleep [%] | 21 | 3 | 19 | 5 | **0.03*** |

# Supplementary Figures


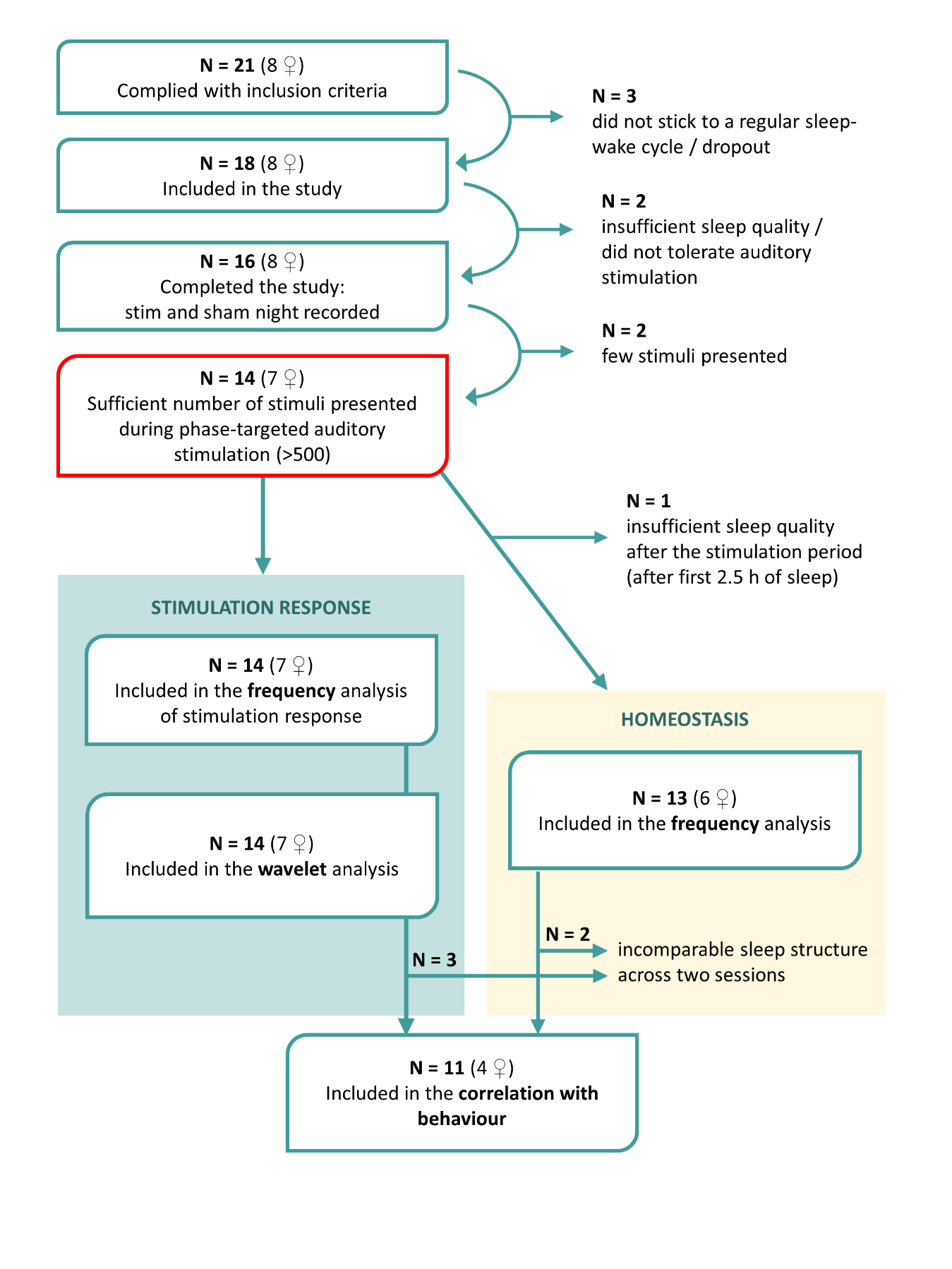


**Supplementary Figure 1.** **Flowchart of exclusion criteria and number of participants included in analyses**.

| 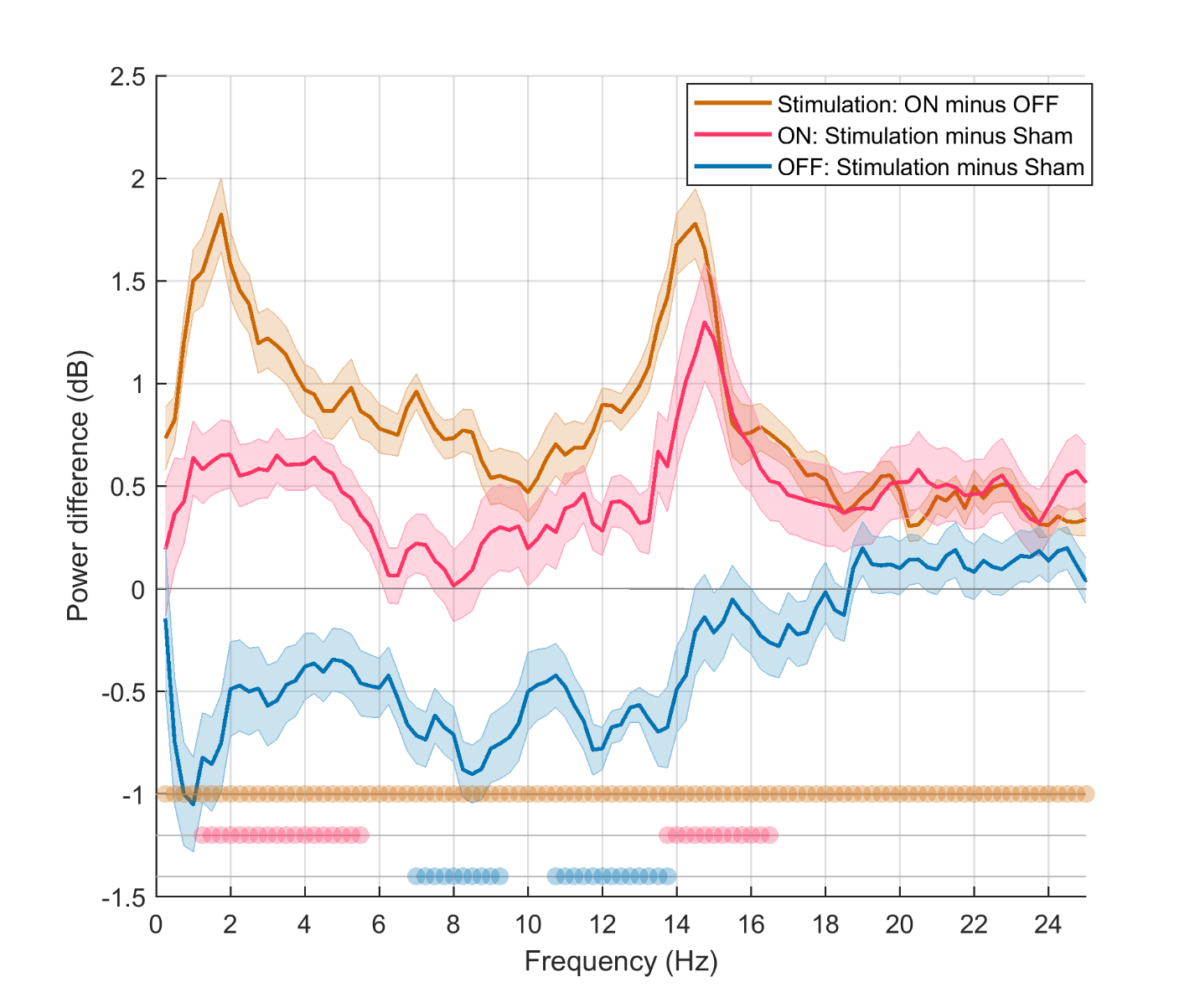 |
| --- |

| **Supplementary Figure 2. Power spectral density change for the within-night and the between-night comparisons.** The data (0.25–25 Hz, divided into 0.25 Hz bins, median ± CI) is presented for the single fronto-centrally located FCz electrode. Thick horizontal bars indicate frequency bins of significant difference between conditions (N=14, cluster corrected paired two-sided t-test, p_clust_<0.05). Note, when comparing ON windows for stim and sham conditions, the increase in delta-theta is accompanied by an increase in sigma power. |
| --- |

| 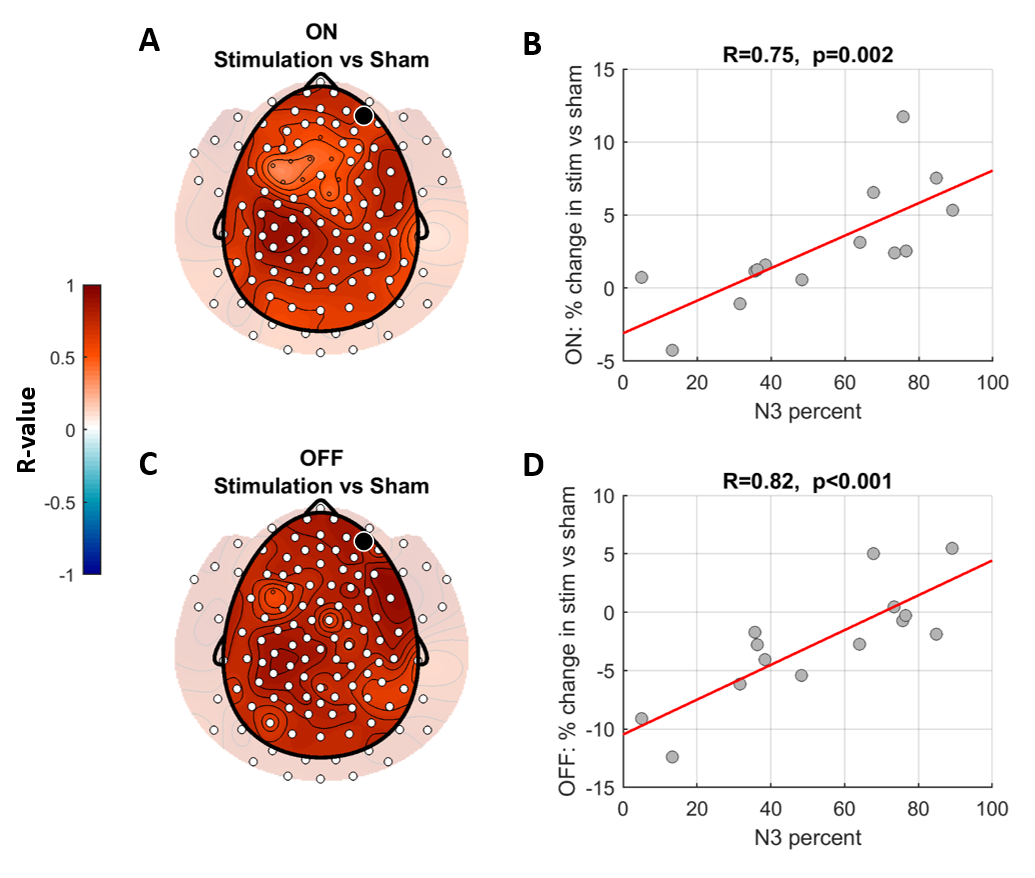 |
| --- |
| **Supplementary Figure 3. Correlation between the stimulation-induced SWA power change and percent of the ON windows belonging to N3 in stimulation condition.** (**A-B**) Topography and scatterplot (average across all channels) of correlation between SWA change in ON windows (between-night contrast of ON windows) and percent of the ON windows belonging to N3 in stimulation condition shows a positive association between these variables. The black dot indicates the target channel Fp2, white dots indicate significant changes (p_clust_<0.05). (**C-D**) Topography and scatterplot of correlation between SWA change in OFF windows (between-night contrast of OFF windows) and percent of N3 showed a similar positive association. |


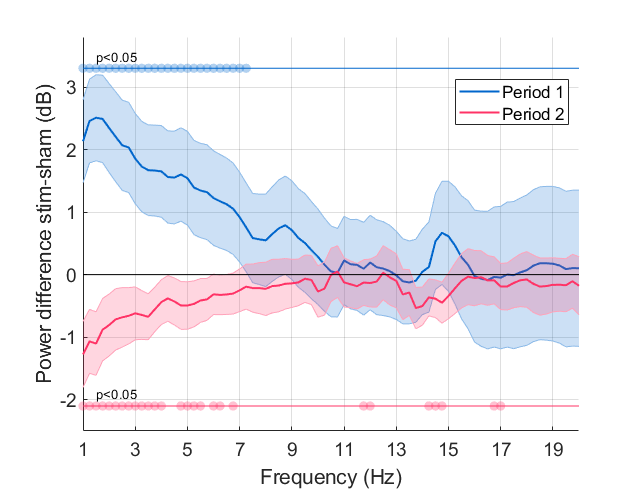


**Supplementary Figure 4. Power density in stimulation (stim) condition normalized to sham (stim-sham) for analysis intervals period 1 and period 2.** The data (1–20 Hz, divided into 0.25 Hz bins, median ± CI) is presented for the single fronto-centrally located FCz electrode. Circles on the horizontal lines indicate frequency bins of significant difference to sham, i.e., zero line (N=13, Wilcoxon rank sum test, p<0.05). 0 dB = 1 µV^2^/Hz.
